# Nasal Obstruction and Mouth Breathing
*"On Nasal Obstruction and Mouth Breathing, as Factors in the Etiology of Caries of the Teeth and in the Development of the Vaulted Palate." By Scanes Spicer, M.D., B.Sc. Pamphlet, 32 pp. (Bale.).


**Published:** 1891-02-07

**Authors:** 


### THE PRACTITIONER'S BOOKSHELF.

### NASAL OBSTRUCTION AND MOUTH BREATHING.*

This is a most excellent paper, containing the results of much original observation. The author throws out hints which should be very valuable, not only to the rhinologist in the treatment of diseases which come under his special care, but also to the dentist. The author's theories seem to give form to much of the observations of past years, and to bear out the experience of daily practice. Certainly, the association of vaulted V-shaped maxillæ, crowded and carious teeth, with enlarged tonsils and nasal troubles, is commonly met with; and the coincidence of the two classes of troubles seems to admit fairly well of Dr. Scanes Spicer's explanation.

* "On Nasal Obstruction and Mouth Breathing, as Factors in the Etiology of Caries of the Teeth and in the Development of the Vaulted Palate." By Scanes Spicer, M.D., B.Sc. Pamphlet, 32 pp. (Bale.)

